# Treatment Outcomes of Clopidogrel in Patients With ACS and Diabetes Undergoing PCI-Analysis of Beijing Municipal Medical Insurance Database

**DOI:** 10.3389/fendo.2021.713849

**Published:** 2021-07-30

**Authors:** Weihao Wang, Xiaoxia Wang, Lina Zhang, Jie Zhang, Fuli Man, Qi Pan, Lixin Guo

**Affiliations:** Department of Endocrinology, Beijing Hospital, National Center of Gerontology, Institute of Geriatric Medicine, Chinese Academy of Medical Sciences, Beijing, China

**Keywords:** clopidogrel, mortality, diabetes, PCI, medical insurance database

## Abstract

**Background and Aims:**

Several clinical trials have proved the efficacy of clopidogrel treatment for patients with percutaneous coronary intervention. There are few large-scale studies to identify the mortality associated with different durations of treatment of clopidogrel in patients with diabetes and ACS undergoing PCI in the Chinese population. The objective of this analysis was to determine the efficacy of long-term clopidogrel therapy (≥12 months) *versus* short-term use (<12 months) in Chinese patients with diabetes after PCI.

**Methods and Results:**

We used the Beijing Municipal Medical Insurance Database provided by the Beijing Municipal Medical Insurance Bureau. The Beijing Municipal Medical Insurance Database contained medical data of about 16 million people, including about 990,000 patients with diabetes and a history of taking antidiabetic medicines. Patients were divided into two groups, one group of 9,116 patients receiving consecutive clopidogrel for one year or more, and another group of 3290 patients receiving consecutive clopidogrel for less than one year. The primary outcomes of this analysis were the risk of all-cause death, myocardial infarction, and revascularization. In patients with diabetes after PCI, long-term clopidogrel treatment was associated with a reduced risk of all-cause death (HR, 0.57[95%CI, 0.49-0.67], P<0.0001), myocardial infarction (HR, 0.79[95%CI, 0.68-0.93], P=0.0035) and an increased risk of angina (HR, 1.18[95%CI, 1.10-1.27], P<0.0001]) and revascularization (HR, 1.07[95%CI, 1.01-1.13], P=0.02]). There was no significant difference in the prevalence of all-cause re-hospitalization, diabetes-related re-hospitalization, and cerebrovascular re-hospitalization.

**Conclusion:**

The present study concluded that long-term dual antiplatelet therapy including clopidogrel and aspirin could decrease the risks of all-cause death, myocardial infarction. But it could increase the risks of angina and revascularization. Further studies should interpret the cause of this question.

## Introduction

Several clinical trials have proved the efficacy of clopidogrel treatment for patients with percutaneous coronary intervention (PCI) (1, 2). For patients with PCI, current guidelines suggest clopidogrel treatment for at least 12 months (3). Non-adherence with clopidogrel after coronary stent implantation could be related to some adverse effects like increased mortality (4). Diabetes is one of the four significant non-communicable diseases and is a major cause of premature death and disability. Among patients with diabetes, the adherence therapy of clopidogrel after myocardial infarction leads to a lower induction in the risk of death (all-cause death and cardiovascular death) (5) compared with it in patients without diabetes. It is well known that patients accompanied by diabetes and acute coronary syndrome (ACS) undergoing PCI are at higher risk for some adverse effects like death (6). The prevalence of diabetes in China has increased 10-fold in the past decade and reached 114 million, making it the country with the highest diabetic population in the world (7, 8). Among Chinese patients with ACS, 37.6% accompanied diabetes or possible diabetes. Even in patients with diabetes younger than 45 years old, 26.9% were accompanied by diabetes or possible diabetes (9). There are few large-scale studies to identify the mortality associated with different durations of treatment of clopidogrel in patients with diabetes and ACS undergoing PCI in the Chinese population.

All citizens with medical insurance are registered with a personal number in China. Since the establishment of China’s medical insurance system, there has been little relevant large-scale clinical data analysis. The Beijing Municipal Medical Insurance Database covers the medical data of thousands of hospitals and community clinics.

In China, the time range of clopidogrel therapy is consistent with guidelines. We conducted large-scale research of 12406 PCI-treated patients with diabetes to evaluate the effect of different durations of treatment of clopidogrel on mortality and other indicators.

## Methods

### Data Source

In China, all medicare citizens are registered with a personal number in the Medical Insurance System. The Beijing Municipal Medical Insurance Bureau holds all information on all outpatients and hospitalizations in Beijing. The Medical Insurance Databse records all the prescriptions and diagnosis information dispensed from hospitals and clinics in Beijing. For calculating the expenses of medicare, all the treatment and use of drugs are registered in the Medical Insurance Databse in China. The study was approved by the ethics committee of Beijing Hospital.

### Population

The population included in this study were all enrolled in the Beijing Municipal Medical Insurance Bureau with available treatment records from 2012-2016. First of all, patients with diabetes diagnoses were selected in Medical Insurance Databse. Then among the patients with diabetes, PCI treatment was identified to locate the patients diagnosed with acute coronary syndrome and diabetes. Patients with survival days of less than 30 days were excluded. Diabetic patients who had at least one time PCI treatment were eligible for further selection. Then patients who have continuous treatment (≥1 year) of aspirin were selected for further investigation in this research. The data extracted from the Medical Insurance Databse contains all medical prescriptions and surgery history.

### Medication Therapy

The Beijing Municipal Medical Insurance Bureau provided medications and diagnoses used from 2012-2016 in patients with diabetes after PCI. Since the drug name appearing in the Medical Insurance Databse may be the chemical name or trade name, we classified the drugs according to the clinical guidelines, such as metformin, sulfonylurea, DPP-4 inhibitors, thiazolidinediones (TZDs), α-glucosidase inhibitors, and glinides (hypoglycemic drugs), diuretics, CCBs, ARB/ACEI, β-receptor inhibitors (antihypertensive drugs). We tracked the dates of prescription of aspirin and clopidogrel up to 4 years after PCI. Patients were considered as not taking aspirin or clopidogrel if the prescription lapsed over 30 days from the last day of the supply. Clopidogrel use was defined as either long-term (≥12 months therapy after PCI) or short-term (<12 months therapy after PCI) therapy.

### Outcomes

Clinical outcomes in patients with diabetes after PCI were identified through the Medical Insurance Databse until December 2016. These outcomes included all-cause death, myocardial infarction, all-cause re-hospitalization, diabetes-related re-hospitalization, cerebrovascular re-hospitalization, angina, and revascularization.

### Statistical Analysis

Quantitative variables were expressed as mean ± SD and categorical variables as frequencies and percentages. Continuous variables with normal distribution were compared between the two groups by the t−test and continuous variables without normal distribution by the Wilcoxon rank−sum test. Categorical variables were compared between groups using the chi-square test or Fisher’s exact probability method (when the expected frequency of cells greater than 25% is less than 5). Events were summarized with Kaplan–Meier curves and estimates at three years. Hazard ratios (HRs) comparing treatment groups were derived from univariate Cox regression models. Subgroups were analyzed with a Cox model, including subgroup, treatment, and the subgroup-by-treatment interaction. Multivariate Cox regression models were derived for the event by the use of a backward selection algorithm. The significance level for staying in the model was set to 0.05 two-sides. All data were prospectively analyzed using SAS, version 9.4.

## Results

### Population Selection

**Figure 1:**
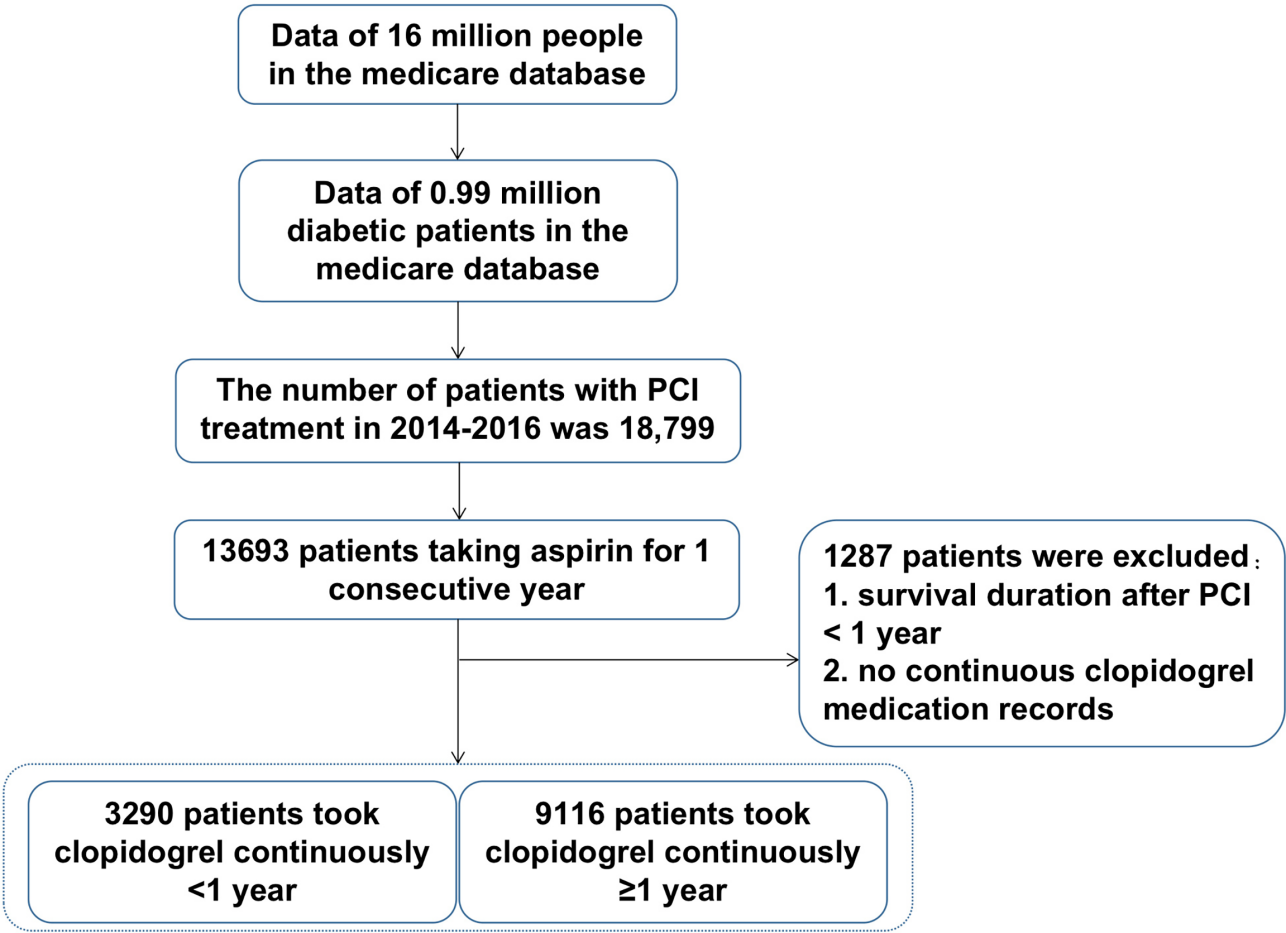
The selection process of included patients.

The selection process was shown in **Figure 1**. The Beijing Municipal Medical Insurance Database contains medical data of about 16 million people, including about 990,000 patients with diabetes and a history of taking antidiabetic medicines. Among them, 18,799 patients had a record of PCI surgery in 2014-2016. Among them, 13,693 patients have continuous aspirin withdrawal records in the database. After excluding patients with survival time of less than one year or no continuous clopidogrel medication withdrawal records, the patients were divided into two groups, one group of 9,116 patients receiving consecutive clopidogrel for one year or more, and another group of 3290 patients receiving consecutive clopidogrel less than one year. All the personal numbers of included participants were masked by desensitization in our research.

### Demographics of Drugs Treatment

We summarized the medication situation of the two groups of patients as shown in **Table 1**. Classifications of drugs contain antidiabetic medicine (Thiazolidinediones, α-glucosidase inhibitors, metformin, sulfonylureas, DPP-4 inhibitors, glinides, insulin), antihypertensive medicine (ARB/ACEI, CCB, β-receptor inhibitors, diuretic), related cardiovascular medicine (statin, nitrate, proton pump inhibitors).

**Table 1:** Demographics of drug treatment.

Drug names	Duration of drug treatment	Nubmer of patients with clopidogrel <1 year (%)	Number of patients with clopidogrel ≥1 year (%)	*P* value
Metformin	none	1089 (33.5)	3032 (33.3)	0.0346
	< 1year	755 (23.2)	1940 (21.3)	
	≥ 1 year	1407 (43.3)	4144 (45.5)	
α-glucosidase inhibitor	none	791 (24.3)	2126 (23.3)	0.1094
	< 1year	723 (22.2)	1925 (21.1)	
	≥ 1 year	1737 (53.4)	5065 (55.6)	
thiazolidinedione (TZD)	none	2878 (88.5)	8076 (88.6)	0.3075
	< 1year	227 (7.0)	679 (7.4)	
	≥ 1 year	146 (4.5)	361 (4.0)	
Sulfonylureas	none	1873 (57.6)	5252 (57.6)	0.4824
	< 1year	559 (17.2)	1496 (16.4)	
	≥ 1 year	819 (25.2)	2368 (26.0)	
Glinides	none	2776 (85.4)	7759 (85.1)	0.8629
	< 1year	265 (8.2)	743 (8.2)	
	≥ 1 year	210 (6.5)	614 (6.7)	
DPP-4 inhibitor	none	3056 (94.0)	8589 (94.2)	0.3037
	< 1year	178 (5.5)	497 (5.5)	
	≥ 1 year	17 (0.5)	30 (0.3)	
insulin	none	1835 (56.4)	5193 (57.0)	0.5542
	< 1year	351 (10.8)	923 (10.1)	
	≥ 1 year	1065 (32.8)	3000 (32.9)	
ticagrelor	none	3122 (96.0)	9029 (99.0)	< 0.0001
	< 1year	114 (3.5)	85 (0.9)	
	≥ 1 year	15 (0.5)	2 (0.1)	
ARB/ACEI	none	499 (15.3)	1463 (16.0)	0.0077
	< 1year	690 (21.2)	1706 (18.7)	
	≥ 1 year	2062 (63.4)	5947 (65.2)	
CCB	none	922 (28.4)	2461 (27.0)	< 0.0011
	< 1year	757 (23.3)	1915 (21.0)	
	≥ 1 year	1572 (48.4)	4740 (52.0)	
β receptor blocker	none	317 (9.8)	883 (9.7)	< 0.0001
	< 1year	566 (17.4)	1190 (13.1)	
	≥ 1 year	2368 (72.8)	7043 (77.3)	
diuretic	none	1802 (55.4)	5110 (56.1)	0.6018
	< 1year	827 (25.4)	2238 (24.6)	
	≥ 1 year	622 (19.1)	1768 (19.4)	
statin	none	11 (0.3)	29 (0.3)	< 0.0001
	< 1year	202 (6.2)	249 (2.7)	
	≥ 1 year	3038 (93.4)	8838 (97.0)	
nitrate	none	377 (11.6)	808 (8.9)	< 0.0001
	< 1year	1248 (38.4)	2841 (31.2)	
	≥ 1 year	1626 (50.0)	5467 (60.0)	
PPI	none	1288 (39.6)	3700 (40.6)	0.0597
	< 1year	1427 (43.9)	3797 (41.7)	
	≥ 1 year	536 (16.5)	1619 (17.8)	

### Mortality and Prevalence of Recurrent Myocardial Infarction and Hospitalization

**Figure 2:**
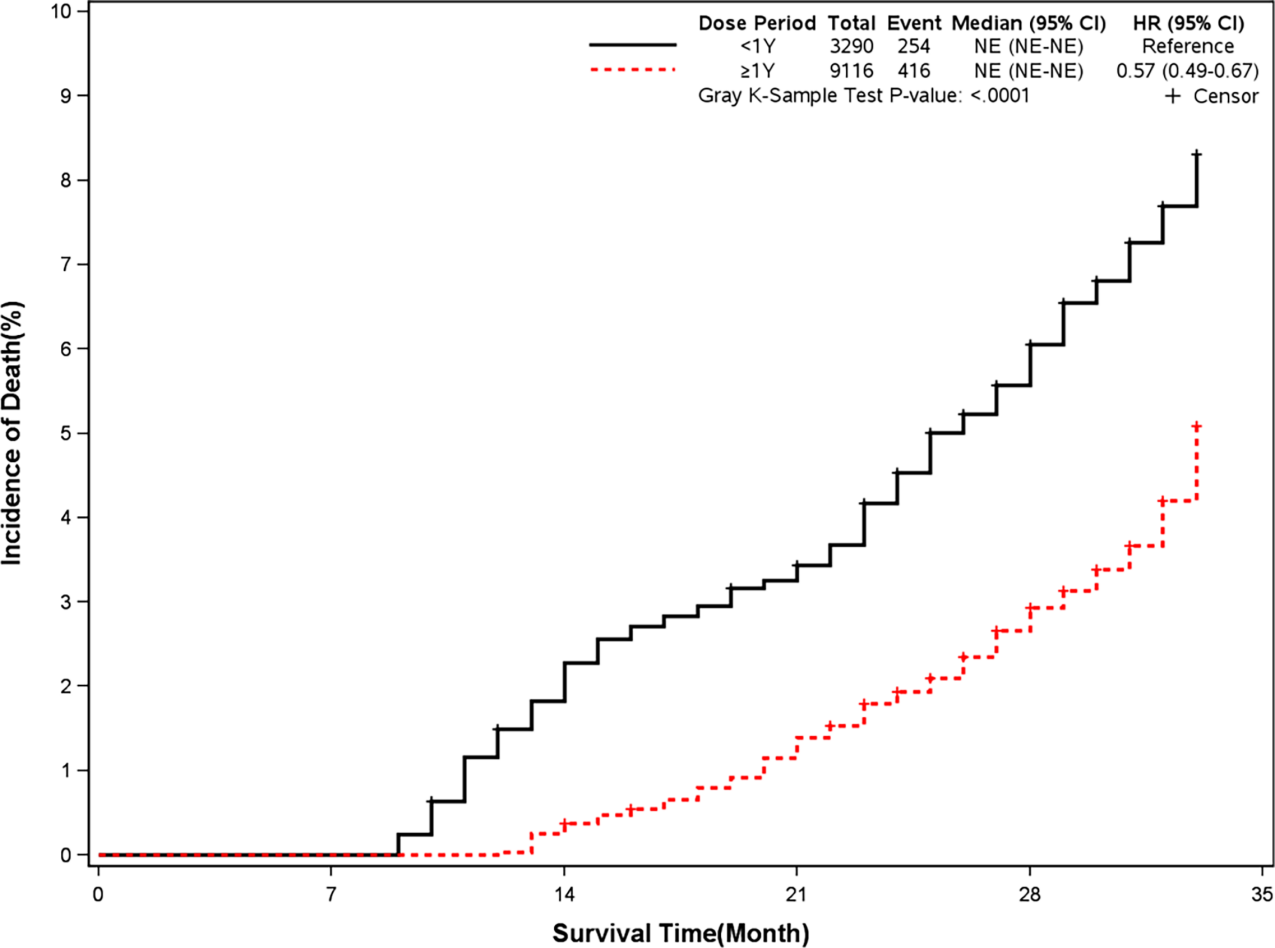
Incidence of all-cause death in two groups. Blackline: patients took clopidogrel continuously < 1 year; Red line: patients took clopidogrel continuously ≥ 1 year.

**Figure 3:**
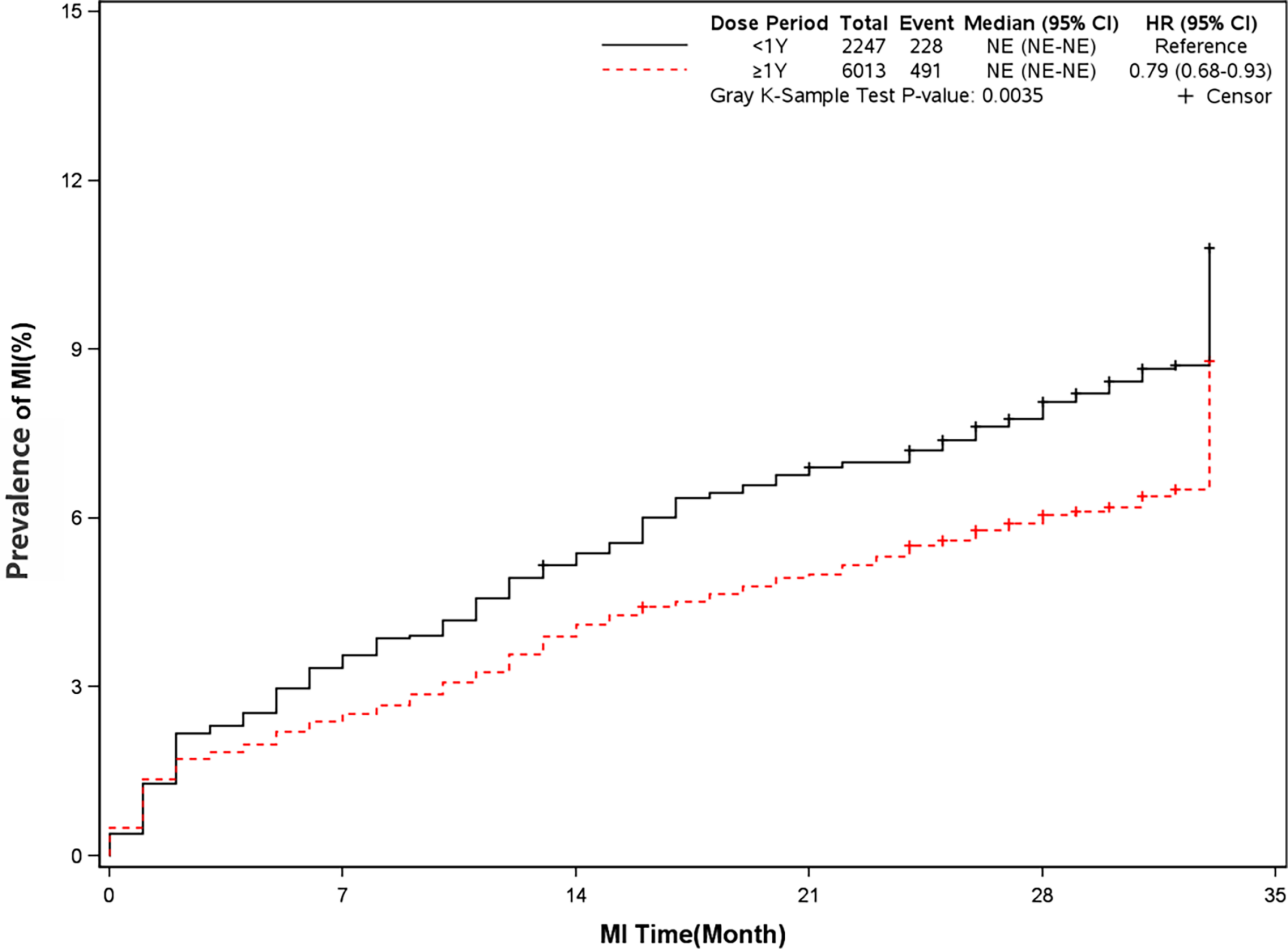
Prevalence of recurrent myocardial infarction in two groups. Blackline: patients took clopidogrel continuously < 1 year; Red line: patients took clopidogrel continuously ≥ 1 year.

**Figure 4:**
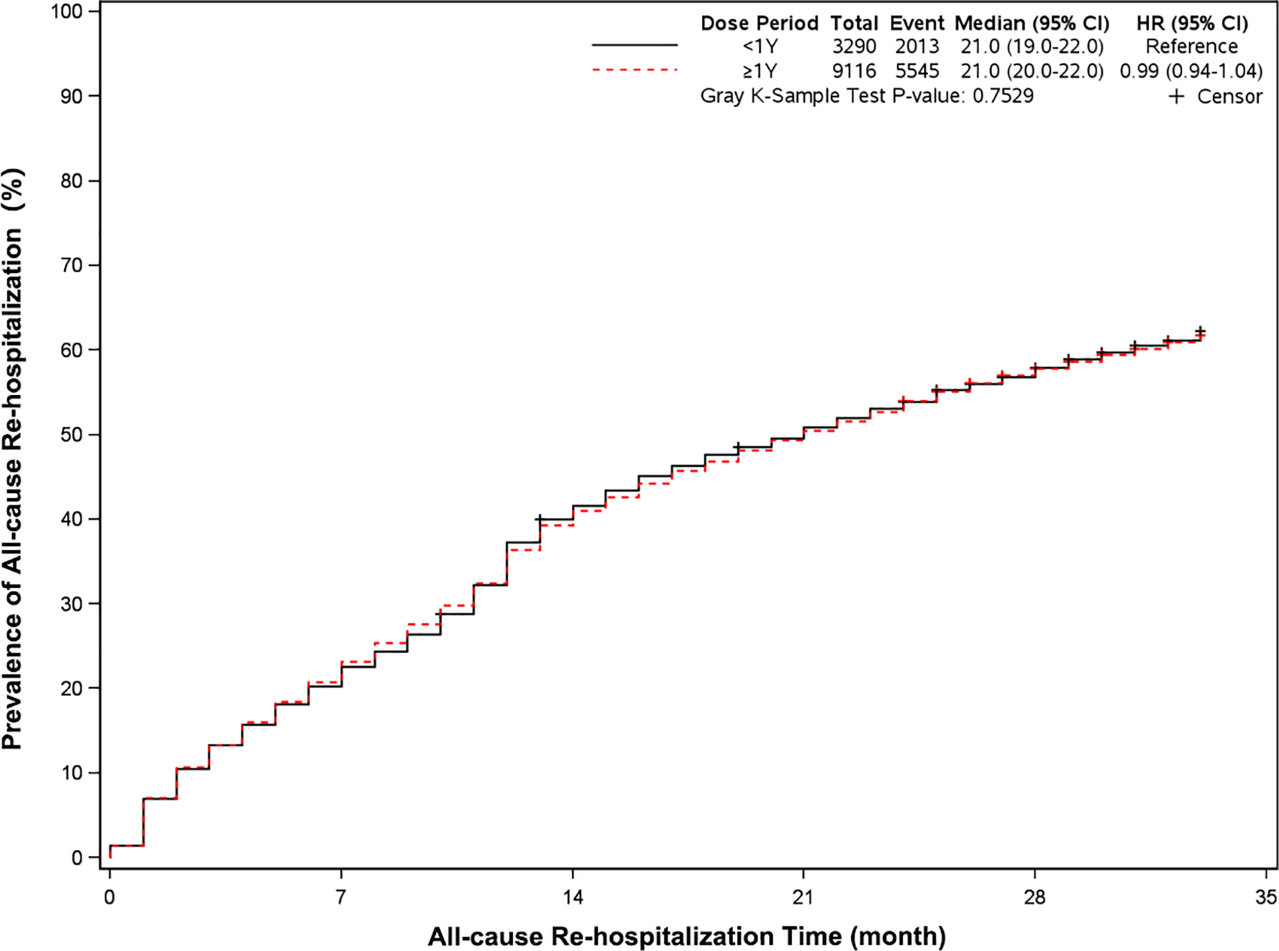
Prevalence of all-cause rehospitalization in two groups. Blackline: patients took clopidogrel continuously 1 year; Red line: patients took clopidogrel continuously ≥ 1 year.

The mortality was lower in patients treated with clopidogrel for more than one year compared with the group treated with clopidogrel less than one year (4.6% *vs* 7.7%, HR, 0.57[95%CI, 0.49-0.67], P<0.0001) (**Figure 2**). The prevalence of myocardial infarction was lower in patients treated with clopidogrel for more than one year compared with patients treated with clopidogrel less than one year (8.2% *vs* 10.1%, HR, 0.79[95%CI, 0.68-0.93], P=0.0035) (**Figure 3**). However, there were no significant differences in the prevalence of all-cause re-hospitalization (P=0.7529), diabetes-related re-hospitalization (P=0.9727), and cerebrovascular re-hospitalization (P=0.2958) (**Figures 4** **–** **6**).

**Figure 5:**
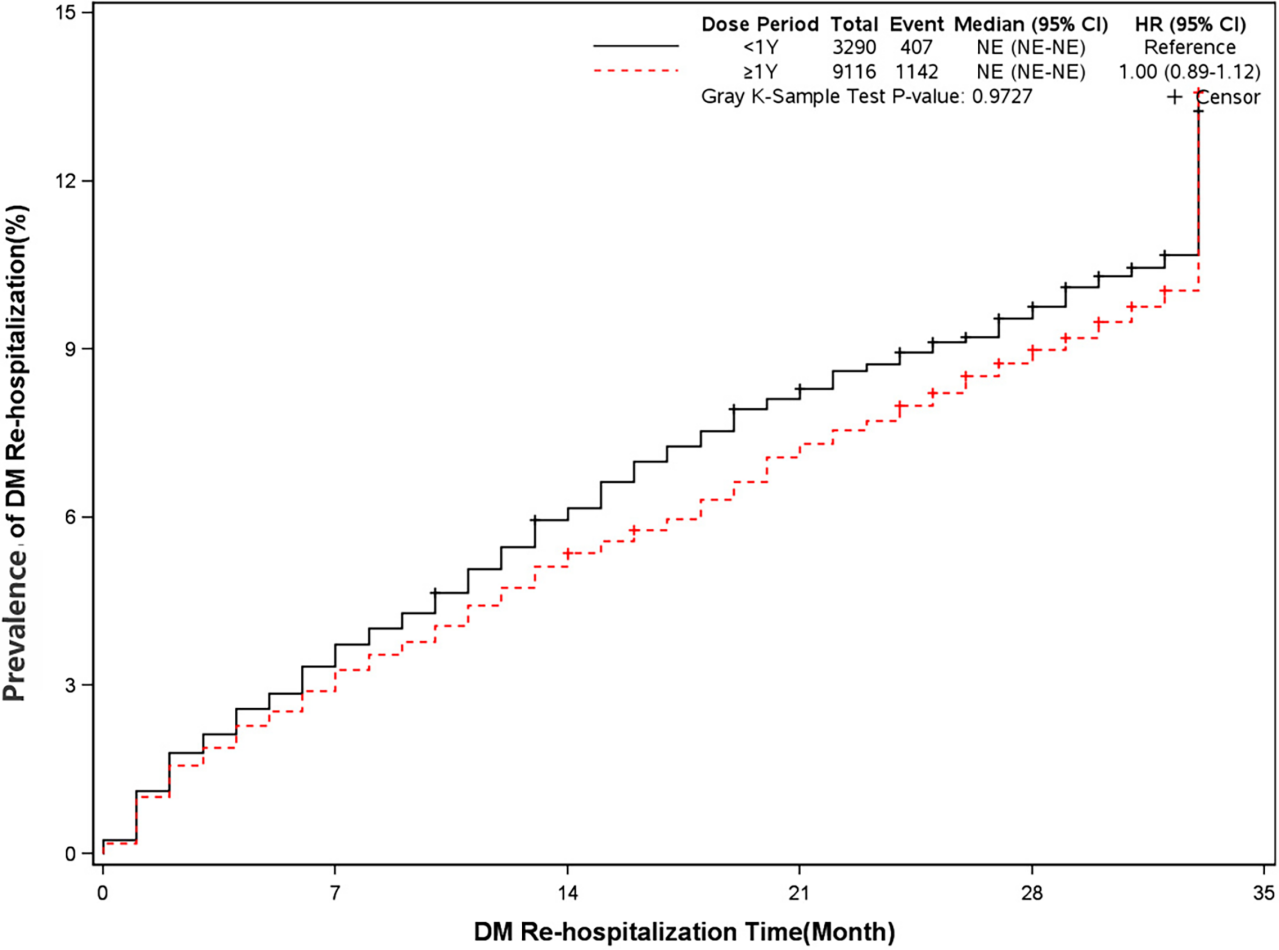
Prevalence of diabetes-related rehospitalization in two groups. Blackline: patients took clopidogrel continuously 1 year; Red line: patients took clopidogrel continuously ≥ 1 year.

**Figure 6:**
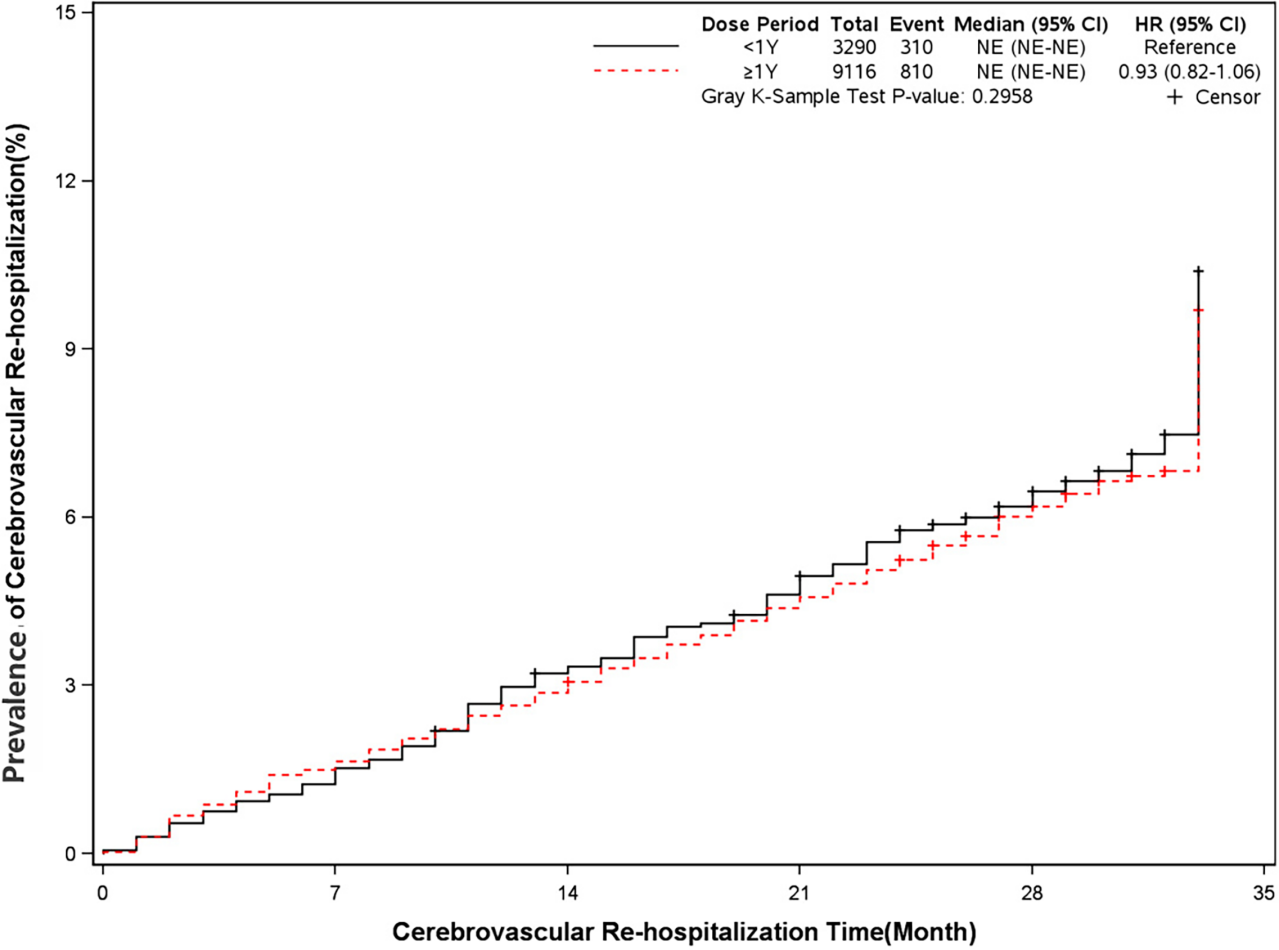
Prevalence of cerebrovascular rehospitalization in two groups. Blackline: patients took clopidogrel continuously < 1 year; Red line: patients took clopidogrel continuously ≥ 1 year.

### Prevalence of Angina and Revascularization

**Figure 7:**
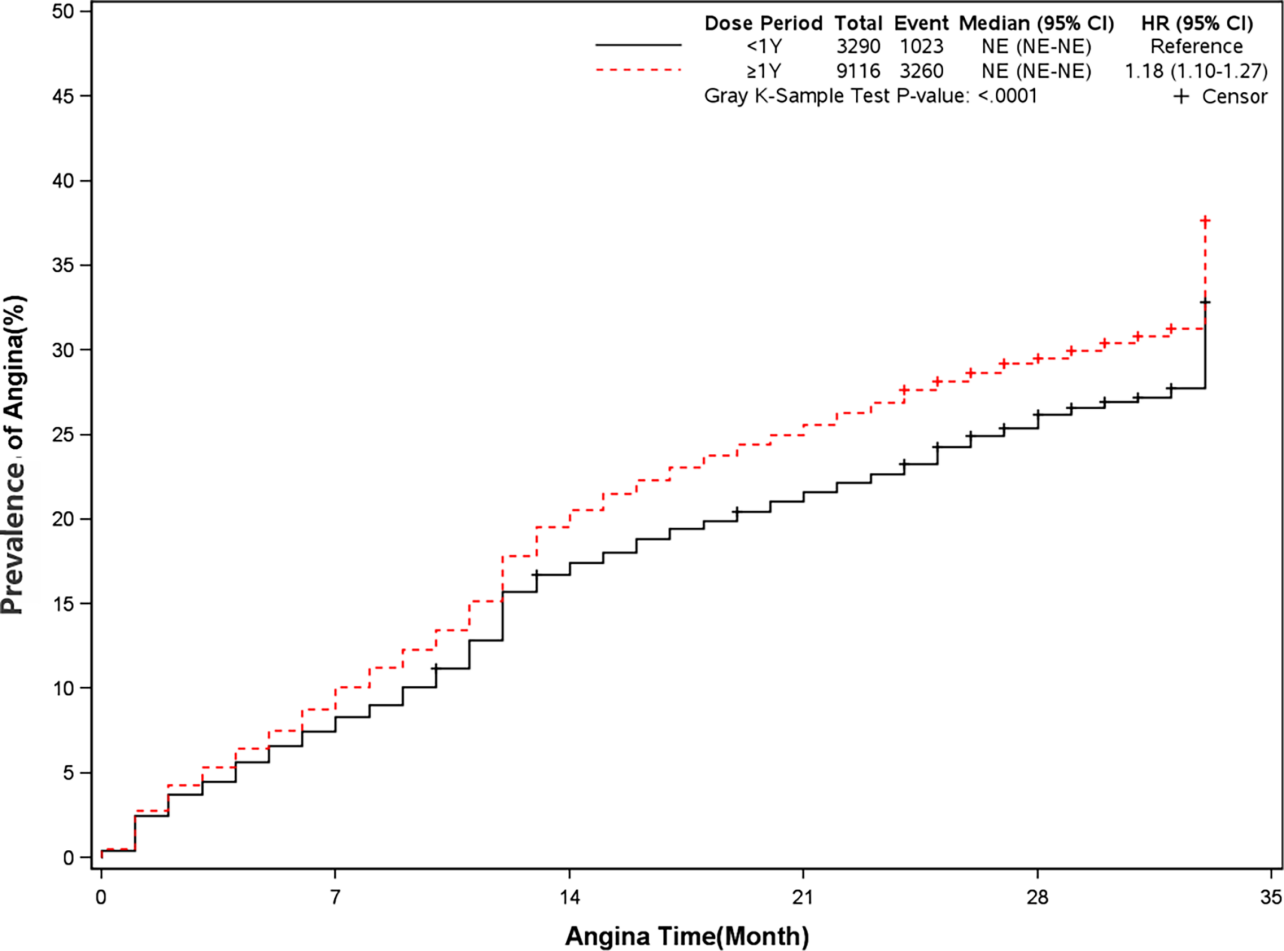
Prevalence of angina in two groups. Blackline: patients took clopidogrel continuously < 1 year; Red line: patients took clopidogrel continuously ≥ 1 year.

**Figure 8:**
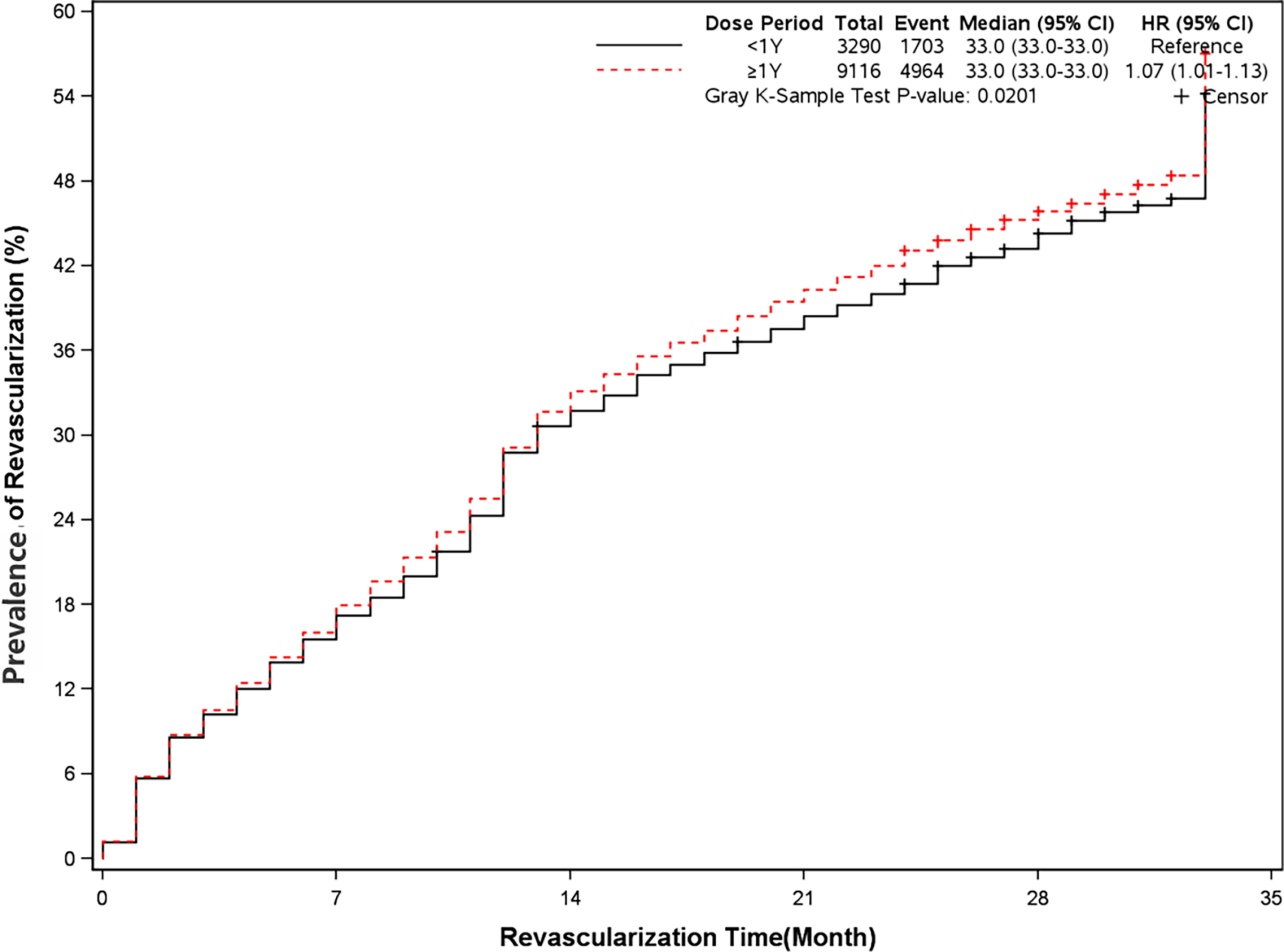
Prevalence of revascularization in two groups. Blackline: patients took clopidogrel continuously < 1 year; Red line: patients took clopidogrel continuously ≥ 1 year.

The rate of angina and revascularization was 35.8% and 54.5% in long-term dual antiplatelet therapy group compared with 31.1% and 51.8% in placebo group (HR, 1.18[95%CI, 1.10-1.27], P<0.0001]) (HR, 1.07[95%CI, 1.01-1.13], P=0.02]) (**Figures 7** and **8**). A long-term combination of aspirin and clopidogrel could cause higher risks of angina and revascularization.

## Discussion

From 2012 to 2016, we screened a cohort of 12,406 patients who underwent PCI surgery among 990,000 diabetic patients in Beijing. The results showed that clopidogrel could reduce all-cause mortality and the probability of recurrent myocardial infarction after more than one year’s regular treatment, but had no significant effect on all-cause readmission, cerebrovascular readmission, and diabetes-related readmission. But long-term dual antiplatelet treatment including aspirin and clopidogrel could increase the risks of angina and revascularization. In the past published articles in the Veterans Health Administration database, in patients with diabetes mellitus who received drug-eluting stents, prolonged clopidogrel (more than 12 months) was associated with a reduced risk of death (10). This result is consistent with the long-term use of clopidogrel in patients without diabetes (4, 11). However, similar studies on the Medical Insurance Database in Chinese population are still lacking.

A high risk of poor clinical outcomes was observed in patients with diabetes after PCI (12, 13). The DAPT study concluded that the treatment of thienopyridine beyond one year could decrease the risks of stent thrombosis, and major cerebrovascular and cardiovascular events (14). As expected, long-term dual antiplatelet therapy could increase the risk of bleeding. That was why our results showed that over 50% of patients had used PPI. However, other research showed that different stents (15) and P2Y12 inhibitors (16) had been related to different rates of stent thrombosis and myocardial infarction. Our research has not focused on this difference as it is inconvenient to distinguish different stents in the China medicare database. The rate of all-cause death and myocardial infarction in the DAPT study was 2.0% and 2.1% in the long-term thienopyridine treatment and 1.5%, 4.1% in the placebo group. But in our study, the rate of all-cause death and myocardial infarction in the long-term dual antiplatelet therapy group was 4.6% and 8.1% compared with 7.7% and 10.1% in the placebo group. This was a big difference between the DAPT study and our results. This result also proved that patients with diabetes after PCI had a higher risk of poor clinical outcomes compared with patients without diabetes. The same aspect between the two studies was that long-term therapy did not affect cerebrovascular outcomes.

Another finding is that long-term dual antiplatelet therapy could increase the risks of angina and revascularization in patients with diabetes undergoing PCI. Controversies still exist with the combination use of clopidogrel and PPI following coronary stenting. The previous meta-analysis (17) showed that the continuous use of clopidogrel and PPI was associated with higher MACES (OR:1.27, 95%CI[1.13-1.42]). Another meta-analysis (18) found that long-term dual therapy leads to increased MACEs, myocardial infarction, stent thrombosis, and target vessel revascularization. Our results showed that 17.8% of patients with long-term dual therapy and 16.5% of patients with short-term dual therapy had used PPI for more than one year. This may be the cause of increased rates of angina and revascularization. The reason for increased adverse effects may be that PPI was involved in the same metabolic pathway (CYP2C19 isoenzyme and so on) as that of clopidogrel (19). However, several studies found different results. From the results of Guthrie Health Off-label Stent (GHOST) research, the combination of PPI and clopidogrel was not related to any increased risk in MACEs outcomes after PCI (20). The specific conclusion still needs further investigation.

Ticagrelor is the first reversibly binding direct P2Y12 inhibitor. It does not need enzymatic activation compared with clopidogrel and prasugrel. It also inhibits platelets faster, better, and more stable than clopidogrel (21, 22). Meanwhile, regardless of revascularization or not, ticagrelor could reduce the risk of all-cause death with no significant increase in the risk of overall bleeding compared with clopidogrel (23). Our results showed that over 95% of diabetic patients after PCI surgery never used ticagrelor. The most possible reason is that ticagrelor has not been included in the medical insurance catalog until 2017 in China.

At the same time, we also summarized the medication situation of patients with diabetes after PCI surgery. We found that the most commonly used antidiabetic drug was alpha-glucosidase inhibitors, followed by metformin. According to the guidelines, most of the lipid-lowering drugs selected for patients with diabetes after PCI were statins (3, 24), which was consistent with our results. More than 90% of patients have used statins for more than one year. But recent research found that treatment with fenofibrate and metformin produces the cardioprotective effect in a rat model with acute myocardial infarction and diabetes (25). And the possible mechanism may act through the PPARα activation.

## Limitations

There was no specific classification of drugs and diseases in the Beijing Municipal Medical Insurance Database. Researchers completed the classification of all drugs and diseases. There were also no basic demographic characteristics and laboratory indicators in the database. It was hard to distinguish the different types of stents to go through further investigation. This study also did not include more potent antiplatelet agents. Although this study was a large-scale investigation, it was only generated from a single city’s database.

## Conclusion

The present study concluded that long-term dual antiplatelet therapy including clopidogrel and aspirin could decrease the risks of all-cause death, myocardial infarction and did not affect the prevalence of all-cause re-hospitalization, diabetes-related re-hospitalization, and cerebrovascular re-hospitalization in patients with diabetes after PCI. But it could increase the risks of angina and revascularization.

## Data Availability Statement

The datasets presented in this article are not readily available because of the policy request. Requests to access the datasets should be directed to the corresponding author LG.

## Ethics Statement

The studies involving human participants were reviewed and approved by Beijing Hospital. Written informed consent for participation was not required for this study in accordance with the national legislation and the institutional requirements.

## Author Contributions 

WW, XW, and LZ made substantial contributions to study design, data collection, data analysis, and manuscript writing. QP and LG made substantial contributions to study design and intellectual direction. JZ and FM made contributions to data collection and analysis. All authors contributed to the article and approved the submitted version.

## Funding

This study was funded by the National Natural Science Foundation of China (Grant No. 81670763 and 81471050).

## Conflict of Interest

The authors declare that the research was conducted in the absence of any commercial or financial relationships that could be construed as a potential conflict of interest.

## Publisher’s Note

All claims expressed in this article are solely those of the authors and do not necessarily represent those of their affiliated organizations, or those of the publisher, the editors and the reviewers. Any product that may be evaluated in this article, or claim that may be made by its manufacturer, is not guaranteed or endorsed by the publisher.
